# Predictors of time to sputum culture conversion in multi-drug-resistant tuberculosis and extensively drug-resistant tuberculosis in patients at Tshepong-Klerksdorp Hospital

**DOI:** 10.4102/sajid.v34i1.111

**Published:** 2019-08-26

**Authors:** Relebohile Ncha, Ebrahim Variava, Kennedy Otwombe, Mary Kawonga, Neil A. Martinson

**Affiliations:** 1 Department of Community Health, School of Public Health, Faculty of Health Sciences, University of the Witwatersrand, Johannesburg, South Africa; 2 Department of Internal Medicine, Klerksdorp-Tshepong Hospital Complex and School of Clinical Medicine, Faculty of Health Sciences, University of the Witwatersrand, Johannesburg, South Africa; 3 Perinatal HIV Research Unit (PHRU), SAMRC Soweto Matlosana Collaborative Centre for HIV/AIDS and TB, University of the Witwatersrand, Johannesburg, South Africa; 4 Centre of Excellence in Biomedical TB Research, University of the Witwatersrand, Johannesburg, South Africa

**Keywords:** Nutrition, Early sputum conversion, Multi-drug-resistant TB, BMI, Predictors, Extensively drug-resistant TB

## Abstract

**Setting:**

Klerksdorp-Tshepong Hospital Complex MDR-TB Unit, North-West Province, South Africa.

**Background:**

To determine the time to sputum culture conversion (TTSCC) and factors predictive of TTSCC in patients with multi-drug-resistant tuberculosis (MDR-TB) and extensively drug-resistant tuberculosis (XDR-TB) in the North-West Province.

**Methods:**

A retrospective cohort study, abstracting patient demographic and clinical data, laboratory results, dates of sputum testing and sputum culture conversion results, from medical records of 526 MDR-TB and 47 XDR-TB patients started on TB treatment between 01 January 2012 and 31 December 2014. Predictors of TTSCC were determined by Cox proportional hazards regression.

**Results:**

The median age was 38 years (interquartile range 31–47) with 64% being male. Overall, 79% (449) were Human Immunodeficiency Virus (HIV)-infected. The median TTSCC was 56.5 days and 162.5 days for MDR-TB and XDR-TB patients, respectively. In the multivariate analysis, age [hazard ratio (HR): 0.89, 95% confidence interval (CI): 0.96–0.99], being underweight (HR: 0.631.61, 95% CI: 0.451.03–0.882.51), Acid Fast Bacilli (AFB) positivity (HR: 0.72, 95 % CI: 0.51–1.01) and having XDR-TB (HR: 0.36. 95% CI: 0.19–0.69) were predictive of longer TTSCC.

**Conclusion:**

Predictors of TTSC allow for MDR-TB- and XDR-TB-diagnosed patients to be identified early for effective management. Those with risk factors for delayed sputum culture conversion which are being underweight and having XDR-TB should be monitored carefully during treatment so that they can achieve sputum culture conversion early.

## Introduction

Multi-drug-resistant (MDR) and extensively drug-resistant (XDR) tuberculosis (TB) are both diseases of global concern. Multi-drug-resistant tuberculosis is defined as resistance to at least both isoniazid and rifampicin, while XDR-TB is defined as resistance to any fluoroquinolone and to at least one of three second-line injectable drugs (capreomycin, kanamycin and amikacin), in addition to multidrug resistance.^1^ According to the World Health Organization (WHO), it is estimated that there were 480 000 new MDR-TB cases and 190 000 deaths globally in 2014.^2^ Drug-resistant TB (DR-TB) is associated with poor TB treatment outcomes; only 52% of MDR-TB patients and 22% of XDR-TB patients were reported cured in the 2013 cohort.^3^

In managing DR-TB, sputum culture conversion from positive to negative is critical in identifying the patient’s response to treatment. Sputum culture conversion is defined as two consecutive negative cultures from sputum samples obtained at least 30 days apart. Earlier sputum culture conversion during DR-TB treatment is associated with more favourable TB treatment outcomes.^4,5^ Differences have been observed between countries with regard to the time to sputum culture conversion (TTSCC) as well as the factors that predict TTSCC. Given the high burden of DR-TB in South Africa, it is important to determine the TTSCC and to understand the factors predictive of the TTSCC among DR-TB patients in South Africa.

According to the WHO Global Tuberculosis Report 2014, South Africa is ranked among the top 10 countries with the highest burden of DR-TB. When looking at the final treatment outcomes, South Africa has poor results. In 2010, only 72% of 7386 diagnosed DR-TB cases were started on treatment, and of these, only 15% were cured, 40% died before completing treatment and 36% were lost to follow up.^6^ In view of the fact that there are variations within and between provinces (geographical regions) in South Africa when it comes to the prevalence of DR-TB and treatment outcome,^7,8^ it is likely that there are differences between provinces in TTSCC and its predictors. It is important that we understand the TTSCC for different provinces to engage with the TB control programmes to tailor relevant interventions for different contexts.

The objective of this study was to determine the TTSCC and factors predictive of TTSCC in patients with MDR-TB and XDR-TB in the North-West Province, one of nine provinces in South Africa.

## Study population and methods

### Study design and setting

This was a retrospective cohort study, where we abstracted clinical and demographic data from medical records of MDR-TB and XDR-TB patients who were 18 years and older and were started on MDR-TB or XDR-TB treatment from 01 January 2012 to 31 December 2014 at the Klerksdorp-Tshepong Hospital Complex MDR-TB Unit in Matlosana North-West Province, South Africa. The province has an estimated population of 3 707 000 people and has an active mining industry (gold and platinum). The province has an MDR-TB prevalence of 2.6% (95%CI: 1.8–3.9) and when further analysed, the MDR-TB prevalence among new TB cases is 1.9% (95%CI: 0.8–3.1) and among previously treated cases, it is 4.3% (95%CI: 1.4–7.1).^8^ When patients are diagnosed with rifampicin resistance TB on GeneXpert, they are referred to the MDR-TB Unit at Klerksdorp-Tshepong Hospital Complex for DR-TB confirmatory tests and further management once MDR/XDR-TB diagnosis is confirmed. This MDR-TB unit is one of two MDR-TB units in the North-West Province and has 76 and 20 MDR-TB and XDR-TB beds, respectively.

### Data collection

Patients who were 18 years and older and were started on MDR-TB and XDR-TB treatment at the Klerksdorp-Tshepong MDR-TB Unit, from January 2012 to December 2014, were eligible for the study. A list of all eligible patients was obtained from the MDR-TB register on site. Medical records for all patients who met inclusion criteria were obtained and reviewed and a structured data extraction tool was used to abstract data. Explanatory variables were patient demographics, clinical characteristics and diagnostic data, TB management data,^1^ sputum conversion data and treatment outcomes data. The outcome variable was TTSCC, defined as the duration, in days, between DR-TB treatment initiation and the date of collection of the first sputum culture specimen that was culture negative after a prior positive culture. Data abstracted to calculate TTSCC were the date of admission (a proxy for date of MDR/XDR-TB treatment initiation) and date of the first sputum that became negative after a previous positive result. Data were captured in Redcap software version 6.17.0.^9^ Approval to conduct this study was obtained from the University of the Witwatersrand Human Research Ethics Committee (Medical), authorisation number: M150639, and permission to conduct the study was obtained from the Klerksdorp-Tshepong Hospital Complex.

### Data analysis

Data were analysed using STATA 14 (*Stata Statistical Software: Release 14*, College Station, TX, USA: StataCorp LP). Continuous demographic and clinical data were described by medians and interquartile ranges (IQRs) and compared by MDR-TB and XDR-TB using the Wilcoxon-sum rank test. Frequencies were determined for categorical variables and compared by MDR-TB and XDR-TB using the chi-square test. Time to sputum culture conversion was determined using the Kaplan–Meier test and the log-rank test which compared the differences between MDR-TB and XDR-TB by various explanatory variables. Predictors of TTSCC were determined by the Cox proportional hazards regression in both the univariate and multivariate analyses. Variables with *p* < 0.2 in the univariate analysis as well as those defined *a priori* were included in the multivariate model. The final model was assessed for the proportional hazards assumption.

## Ethical consideration

Ethical Clearance was issued on 26 June 2015 by the University of Witwatersrand Human Research Ethics Committee (Medical), number M150639.

## Results

A total of 573 patients were enrolled in the study. The median age was 38 years (IQR 31–47), similar between MDR-TB and XDR-TB patients (*p* = 0.943). There were statistically significant differences between MDR-TB and XDR-TB patients for employment (43% vs 70%, *p* < 0.001). However, there were no differences between MDR-TB and XDR-TB patients – with formal education (89% vs 82%, *p* = 0.188), or their smoking history (26% vs 21%, *p* = 0.475), using alcohol (26% vs 35%, *p* = 0.249) or having an occupational history in gold mining (8% vs 13%, *p* = 0.234) (Table 1). The median body mass index (BMI) was 18.1 (IQR: 16.2–21.2) and was similar between MDR-TB and XDR-TB patients (*p* = 0.521). Of the 565 participants tested for HIV, 448 (79%) were HIV-infected and of those with an available baseline CD4 count results on admission (430/448), 402 (93%) had a CD4 count < 500 cells/mm^3^. Of the 448 who were HIV-positive, 257 (58%) were on antiretroviral therapy (ART) on admission to hospital. There were no significant differences between MDR-TB and XDR-TB patients for their median haemoglobin (11.05 [IQR: 9–13] vs 11.8 [IQR: 9.9–13.4], *p* = 0.134), creatinine concentration (66 [IQR: 55–80] vs 71 [IQR: 62–87], *p* = 0.083) as well as CD4 counts (119 [IQR: 52–255] vs 113 [IQR: 44–252], *p* = 0.820), as seen in Table 2.

**TABLE 1:** Demographic characteristics of the participants.

Variables	Overall		MDR-TB		XDR-TB		*p*
	*n*	%	*n* (526)	% (92)	*n* (47)	% (8)	
**Demographic characteristics**							
Age (years) median (IQR)	38	31–47	38	31–47	37	32–47	-
**Race**							
Black	568	99	522	99	46	98	0.334
**Gender**							
Male	366	64	333	63	33	70	0.345
**Employment status**							
Employed	261	46	228	43	33	70	< 0.001
**Education status** †							
Formal schooling	474	88	437	89	37	82	0.188
**Smoking history†**							
Yes	101	26	94	26	7	21	0.475
**Alcohol history†**							
Yes	105	27	93	26	12	35	0.249
**Gold mining history**							
Yes	47	8	41	8	6	13	0.234

MDR-TB, multi-drug-resistant tuberculosis; XDR-TB, extensively drug-resistant tuberculosis; IQR, interquartile ranges.

† , Variables have missing data.

**TABLE 2:** Clinical characteristics of the participants.

Variables	Overall		MDR-TB		XDR-TB		*p*
	*n*	%	*n* (526)	% (92)	*n* (47)	% (8)	
**Clinical characteristics**							
BMI (kg/m^2^) median (IQR)	18.1	16.2–21.2	18.1	16.1–21.1	18.1	16.7–21.6	0.521
Haemoglobin (g/dL) median (IQR)	11.1	9.1–13	11.05	9–13	11.8	9.9–13.4	0.134
Creatinine (mg/dL) median (IQR)	67	55–81	66	55–80	71	62–87	0.083
CD4 count (cells/mm^3^)	118	51–255	119	52–255	113	44–252	0.82
**BMI (kg/m^2^)†**							
Normal weight	87	38	80	38	7	37	0.927
Overweight	19	8	17	8	2	11	
Underweight	126	54	116	54	10	53	
**Past TB history**							
Yes	345	60	314	60	31	66	0.401
**X-ray cavities†**							
None	340	61	315	62	25	53	0.395
Unilateral	165	30	147	29	18	38	
Bilateral	52	9	48	9	4	9	
**HIV status**							
Positive	448	79	407	79	41	87	0.161
**ART use at admission†**							
Defaulted	47	10	45	11	2	5	0.464
Naïve	142	32	128	32	14	34	
On treatment	257	58	232	57	25	61	
**CD4 count (cells/mm^3^)†**							
< 500	402	93	369	94	4	11	0.268
> 500	28	7	24	6	33	89	
**Hypertension**							
Yes	31	5	29	6	2	4	0.715
**Diabetes**							0.656
Yes	8	1	7	1	1	2	

TB, tuberculosis; MDR-TB, multi-drug resistant tuberculosis; XDR-TB, extensively drug-resistant tuberculosis; IQR, interquartile ranges; ART, antiretroviral therapy; BMI, body mass index; cluster of differentiation 4 count (CD4 count).

† , These variables have missing data.

Among those enrolled, 355 (62%) had a GeneXpert MTB/RIF (GXP) result prior to initiation of therapy of whom 352 (99%) were positive for *Mycobacterium tuberculosis* with rifampicin resistance. Of those with a culture result on admission, 374 (87%) had a positive sputum culture for *M. tuberculosis*. Of those enrolled, 526 (92%) were diagnosed at baseline with MDR-TB and 47 (8%) with XDR-TB. Of most patients, 271 (38%) had treatment success, 48 (8%) defaulted treatment, 137 (24%) died and 171 (30%) were transferred out (Table 3).

**TABLE 3:** Drug-resistant tuberculosis diagnosis and treatment outcome data of participants.

Variables	Overall		MDR-TB		XDR-TB		*p*
	*n*	%	*n* (526)	% (92)	*n* (47)	% (8)	
**Diagnosing TB**							
***Sputum smear*†**							
Negative	206	41	193	42	13	29	0.186
Positive	295	59	263	58	32	71	
***GXP performed?*†**							
No	217	38	191	36	26	55	0.010
Yes	355	62	334	64	21	45	
***GXP results*†**							
Negative	3	1	3	1	0	0	-
Positive	352	99	331	99	21	100	
***GXP+ Rif resistant*†**							
No	1	1	1	1	0	0	-
Yes	351	99	330	99	21	100	
***Culture results*†**							
Negative	56	13	56	14	0	0	-
Positive	371	83	329	82	42	98	
Inconclusive	18	4	17	4	1	2	
**DR TB treatment outcomes**							
***Treatment outcome***							
Treatment success	217	38	203	39	14	30	0.256
Deceased	137	24	127	24	10	21	
Defaulted	48	8	45	9	3	6	
Other	171	30	151	29	20	43	

TB, tuberculosis; MDR-TB, multi-drug-resistant tuberculosis; XDR-TB, extensively drug-resistant tuberculosis; DR-TB, drug-resistant tuberculosis; GXP, GeneXpert.

† , These variables have missing data.

**FIGURE 1:**
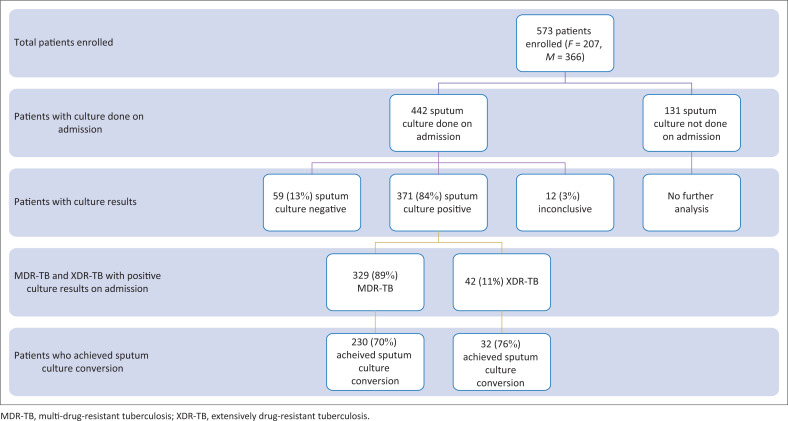
Number of participants included in the analysis of time to sputum culture conversion.

The final analysis of the TTSCC used data from 371 patients with a positive sputum culture result on admission. Of these, 329 (89%) were MDR-TB patients, while 42 (11%) were XDR-TB patients (Figure 1).

**FIGURE 2:**
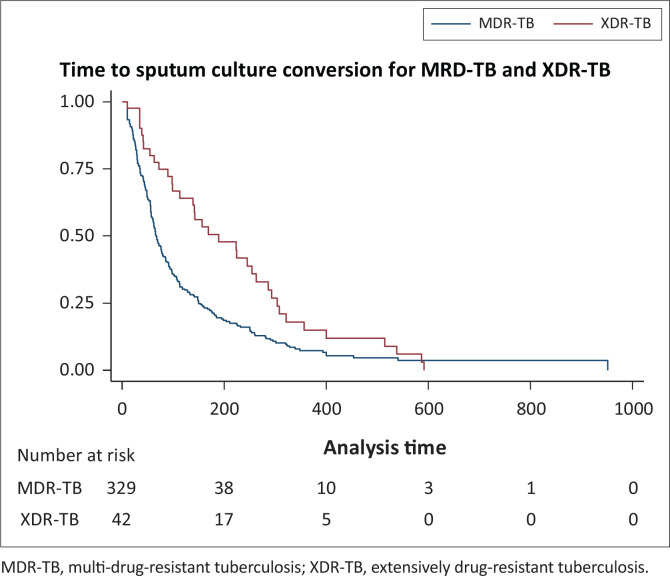
Time to sputum culture conversion for multi-drug-resistant tuberculosis and extensively drug-resistant tuberculosis

The overall median TTSCC for both the MDR-TB and XDR-TB patients was 58.2 days (IQR 29–113); and 56 days (IQR 28–99) and 139 days (IQR 54–245) for MDR-TB and XDR-TB patients, respectively (Figure 2). A total of 262/374 (70%) achieved sputum culture conversion. Of these, 230 (69%) were MDR-TB patients and 32 (76%) were XDR-TB patients. Of the patients who achieved sputum culture conversion, 16 (50%) XDR-TB patients were treated using 8–10 drugs and 13 (41%) of the XDR-TB patients converted between 61 and 180 days, while 196 (75%) of MDR-TB patients were treated using three to five drugs and 138 (53%) converted in less than 60 days. Of the 230 MDR-TB patients who achieved sputum culture conversion, 111 (48%) achieved treatment success and 35 (15%) died; while among the 32 XDR-TB patients, 13 (41%) achieved treatment success and 3 (9%) died (Table 4).

**TABLE 4:** Drug-resistant tuberculosis, time to sputum culture conversion, total number of drugs and treatment outcome for patients who achieved sputum culture conversion.

Variables	Overall		MDR-TB		XDR-TB		*p*
	*n*	%	*n* (230)	% (88)	*n* (32)	% (12)	
**TTSCC (in days)**							
< 60 days	138	53	129	56	9	28	<0.001
61–180 days	91	35	78	34	13	41	
> 180 days	33	12	22	10	11	31	
**Total number of drugs used**							
3–5 drugs	196	75	193	84	3	9	-
6–7 drugs	50	19	37	16	13	41	
8–10 drugs	16	6	0	0	16	50	
**Treatment outcome**							
Treatment success	124	47	111	48	13	41	-
Deceased	38	15	35	15	3	9	
Defaulted	13	5	12	5	1	3	
Other	87	33	72	31	15	47	

TTSCC, time to sputum culture conversion; MDR-TB, multi-drug-resistant tuberculosis; XDR-TB, extensively drug-resistant tuberculosis.

In the univariate analysis model, being underweight (HR: 0.68, 95% CI: 0.49–0.94, *p* = 0.023), Acid Fast Bacilli (AFB) positivity on admission (HR: 0.71, 95% CI: 0.56–0.88, *p* = 0.002) and having XDR-TB (HR: 0.51, 95% CI: 0.36–0.73, *p* < 0.001) were associated with longer TTSCC (Table 5).

**TABLE 5:** Univariate and multivariate analysis of predictors of time to sputum culture conversion.

Variable	Univariate analysis			Multivariate analysis		
	Hazard ratio	95% CI	*p*	Hazard ratio	95% CI	*p*
**Age per 10 year increase (years)**	0.99	0.98–1.00	0.137	0.98	0.96–0.99	**0.024**
**Gender**						
Female	ref	-	-	-	-	-
Male	0.94	0.76–1.14	0.525	-	-	-
**Employment status**						
Unemployed	ref	-	-	ref	-	-
Employed	0.88	0.72–1.06	0.190	1.28	0.87–1.57	0.200
**Education status**						
No formal education	ref	-	-	ref	-	-
Formal education	1.36	0.98–1.88	0.067	1.83	0.83–4.02	0.128
**Smoking history**						
No	ref	-	-	-	-	-
Yes	0.81	0.61–1.07	0.138	0.75	0.40–1.40	0.377
**Alcohol history**						
No	ref	-	-	-	-	-
Yes	0.81	0.61–1.06	0.122	1.07	0.63–2.54	0.488
**Gold mining history**						
No	ref	-	-	-	-	-
Yes	1.15	0.82–1.62	0.408	-	-	-
**BMI categories (kg/m^2^)**						
Normal weight	ref	-	-	ref	-	-
Underweight	0.68	0.49–0.94	**0.023**	0.63	0.45–0.88	**0.008**
Overweight	0.97	0.55–1.71	0.921	1.08	0.61–1.91	0.779
**CD4 count categories (cell/mm^3^)**						
> 500	ref	-	-	-	-	-
< 500	0.99	0.61–1.61	0.967	-	-	-
**Haemoglobin (g/dL)**	1.01	0.99–1.03	0.128	1.06	0.96–1.17	0.235
**Creatinine (mg/dL)**	1	1.00–1.01	**<0.001**	1.00	0.99–1.01	0.169
**AFB count**						
Negative	ref	-	-	-	-	-
Positive	0.71	0.56–0.88	**0.002**	0.72	0.51–1.01	0.059
**Hypertension**						
No	ref	-	-	-	-	-
Yes	0.89	0.59–1.35	0.596	-	-	-
**Diabetes**						
No	ref	-	-	-	-	-
Yes	0.91	0.41–2.04	0.827	-	-	-
**X-ray cavities**						
None	ref	-	-	-	-	-
Unilateral	0.85	0.67–1.05	0.139	1.21	0.81–1.79	0.336
Bilateral	0.81	0.54–1.15	0.250	0.92	0.51–1.67	0.804
**Past TB history**						
No	ref	-	-	-	-	-
Yes	1.01	0.83–1.24	0.861	-	-	-
**HIV status**						
Negative	ref	-	-	-	-	-
Positive	1.04	0.82–1.31	0.741	-	-	-
**ARV status**						
On treatment	ref	-	-	-	-	-
Naive	0.87	0.68–1.12	0.311	-	-	-
**DR-TB diagnosis**						
MDR-TB	ref	-	-	ref	-	-
XDR-TB	0.51	0.36–0.73	**< 0.001**	0.36	0.19–0.69	**0.002**

Note: The numbers in bold represent variables with *p*-values that are statistically significant.

DR-TB, drug-resistant tuberculosis; MDR-TB, multi-drug-resistant tuberculosis; XDR-TB, extensively drug-resistant tuberculosis; AFB, acid fast bacilli; ARV, antiretroviral treatment.

In the multivariable analysis that included *a priori* variables, being underweight (HR: 0.63, 95% CI: 0.45–0.88, *p* = 0.008), having XDR-TB (HR: 0.36, 95% CI: 0.19–0.69, *p* = 0.002) and older age (HR: 0.98, 95% CI: 0.96–0.99, *p* = 0.024) were associated with longer TTSCC. AFB positivity on sputum on admission was marginally significant and was associated with longer TTSCC (HR: 0.72, 95% CI: 0.51–1.01, *p* = 0.059) (Table 5).

## Discussion

Early sputum culture conversion is predictive of favourable TB treatment outcomes.^10^ This study reports the TTSCC and its predictors among MDR-TB and XDR-TB patients treated at Klerksdorp-Tshepong Hospital in South Africa. We found the median TTSCC for MDR-TB and XDR-TB patients to be 56 days (IQR 28–99) and 139 days (IQR 54–245), respectively. Being underweight, AFB positivity on admission and being diagnosed with XDR-TB were associated with longer TTSCC. Our study was not able to demonstrate predictors of shorter TTSCC.

Our findings regarding TTSCC were similar to those found in a KwaZulu-Natal study which reported a median TTSCC of 62 days (IQR: 48–111) for MDR-TB patients;^11^ but different from studies in three other provinces in South Africa (Cape Town, North West and Gauteng) which reported significantly longer (> 270 days) TTSCC for XDR-TB patients.^12^ These results are in keeping with what was found in Peru, where the XDR-TB patients obtained culture conversion later than MDR-TB patients.^13^

Evidence has shown that several factors influence both shorter and longer TTSCC. Factors that influence longer sputum conversion include the drug-resistance pattern on drug susceptibility testing (DST), a high baseline sputum bacilli count, patients with cavities on the chest X-ray and patients with a previous history of susceptible TB or MDR-TB with a negative outcome such as treatment failure or relapse, while factors associated with shorter conversion include being HIV co-infected.^14,15,16,17,18,19^ In our study, patients who were underweight achieved longer TTSCC than those with a normal weight and this was also found in the literature.^20^ In our study, most patients were underweight on admission. Compared to those with normal weight, MDR-TB patients who are underweight are likely to present with clinical characteristics identified as predictors of longer TTSCC, including severe disease, cavities on the X-ray and a high sputum bacilli count.^16,17,21,22^ In the literature, the causal relationship between being underweight and DR-TB has not been proven but we do know that DR-TB can lead to wasting, increased risk of DR-TB and death.^20,23^ Underweight patients have macro- and micronutrient deficiencies which impair immune response to infections.^24^ Although the causal relationship between weight gain and shorter TTSCC has not been proven, studies have shown that being underweight can lead to unfavourable treatment outcomes such as treatment failure and death. Patients who are underweight have reduced bioavailability and malabsorption of anti-TB drugs and this can contribute to longer TTSCC and subsequently treatment failure.^21,24^ A randomised control trial in India showed that adding nutritional supplementation can lead to a higher sputum conversion rate.^25^ Therefore, patients with DR-TB who are older and underweight should be assessed for hypoalbuminemia and micronutrient deficiencies. Nutritional supplements should be provided as studies support shorter TTSCC.^23,25,26,27^

Our findings have shown that patients with XDR-TB are likely to take longer to achieve sputum culture conversion compared to MDR-TB patients. Extensively drug-resistant TB is resistant to the most potent anti-TB drugs (rifampicin and isoniazid) as well as second-line drugs and injectables and fluoroquinolones and this makes the treatment of XDR-TB a challenge. The combination therapy for XDR-TB consists of less potent, highly toxic anti-TB drugs which are given over a longer period and this contributes to longer TTSCC.^28^ During our study, new drugs such as bedaquiline and delaminid were undergoing trials which have shown that the use of bedaquiline can shorten the TTSCC in treatment of DR-TB.^29^ Therefore, the inclusion of such drugs in the management of patients with MDR-TB and XDR-TB can lead to shorter TTSCC.

Our study confirmed that AFB positivity on the smear confirmed findings from previous studies.^17,20,30^ High bacilli count on admission demonstrates the high burden of resistant bacilli in the lung. Second-line drugs that are used to treat DR-TB are less efficacious, the bacilli will take longer to respond to the treatment and this leads to longer TTSCC.^7^ Our study demonstrated that increased age correlates with longer TTSCC. Patients develop more co-morbid diseases and have reduced immune responses and more drug–drug interactions as they age. These factors may also increase TTSCC.^31^

The study has several limitations. Retrospective record reviews are limited by missing or incomplete data, and for our study, we had missing data such as the specific date of treatment initiation; therefore, we used the data of admission as a proxy for date of treatment initiation. There were missing follow-up sputum culture results as well and this influenced the number of censored patients in the survival analysis as well as the confirmation of cured patients. The impact of missing data was also evident with regard to HIV tests, CD4 count and Antiretroviral (ARV) treatment. The denominator for the CD4 count is different from the total number of HIV-positive patients and the denominator for patients on ARVs on admission is different from the number of patients who are HIV-positive and had a CD4 count.

## Conclusion

The time to sputum culture conversion has both clinical and public health significance in managing DR-TB. Longer TTSCC is an indicator for poor DR-TB treatment outcomes, such as treatment failure, relapse and death. Our study has shown that age, being underweight while on DR-TB treatment, AFB positivity on admission and having XDR-TB predict longer TTSCC. Although the causal relationship between weight gain and shorter TTSCC has not been proven, studies have shown that being underweight can lead to unfavourable treatment outcomes such as death. Patients with AFB positivity should be monitored closely and their treatment optimised as they are infectious and are likely to spread the disease further.

## Footnotes

1 List of anti-TB drugs: Group 1: First line drugs: Ethambutol, Pyrazinamide; Group 2: Injectable: Kanamycin, Amikacin, Capeomycin, Viomycin; Group3: Flouroquinolones: Levofloxacin, Moxifloxacin; Group 4: second line drugs: Ethionamide, Prothionamide, Cycloserine, Terizidone, PAS; Group 5: Other.
